# Exploring the potent enhancement effects of plyometric training on vertical jumping and sprinting ability in sports individuals

**DOI:** 10.3389/fphys.2024.1435011

**Published:** 2024-09-06

**Authors:** Lin Xie, Jiayong Chen, Jing Dai, Wenfeng Zhang, Lunxin Chen, Jian Sun, Xiang Gao, Junyi Song, Hailun Shen

**Affiliations:** ^1^ Digital Physical Training Laboratory, Guangzhou Sport University, Guangzhou, China; ^2^ School of Athletic Training, Guangzhou Sport University, Guangzhou, China; ^3^ Zhejiang Technical Institute of Economics, Hangzhou, China

**Keywords:** plyometric training, post-activation potentiation, post-activation performance enhancement, explosive power, sprint

## Abstract

**Objective:**

This meta-analysis examines the impact of different combinations of plyometric training (complexity, training volume, and rest intervals) on immediate vertical jump and sprint performance in athletes.

**Methods:**

A systematic search was conducted in four databases, and Cochrane guidelines were used to evaluate the quality of included studies. Review Manager 5.4 software was employed to analyze outcome measures. Nineteen randomized controlled trials involving 293 participants were included.

**Results:**

Single plyometric training-induced post-activation potentiation (PAP) had a slight positive effect on vertical jump performance [SMD = −0.24, 95% CI (−0.38, −0.1), *P* = 0.0009]. Optimal results were observed with rest intervals of 0.3–4 min (SMD = 0.30, *P* = 0.0008). Sprint performance showed slight improvement [SMD = 0.27, 95% CI (0.03, 0.52), *P* = 0.03]. Complex plyometric training had a moderate effect on vertical jump performance [SMD = 0.58, 95% CI (−0.86, −0.23), *P* = 0.002], with the best outcomes seen with rest intervals exceeding 8 min (SMD = 0.77). Sprint performance also improved significantly [SMD = 0.8, 95% CI (0.01, 1.59), *P* = 0.05]. Single-session plyometric training did not significantly enhance vertical jump performance [SMD = −0.19, 95% CI (−0.41, −0.02), *P* = 0.07], but had a notable effect on sprint performance [SMD = 0.8, 95% CI (0.01, 1.59), *P* = 0.05], particularly with rest intervals exceeding 8 min (SMD = 0.77). Multiple-session plyometric training improved vertical jump (SMD = 0.43, 95% CI [0.01, 1.59), *P* = 0.00001 < 0.05], with optimal effects observed at rest intervals of 5–7 min (SMD = 0.64). Sprint performance also improved [SMD = 0.46, 95% CI (0.01, 0.81), *P* = 0.01 < 0.05].

**Conclusion:**

Plyometric training as an activation method has significant enhancing effects, depending on training complexity, volume, and rest intervals.

## Introduction

Lower limb explosive strength plays a crucial role in numerous athletic disciplines and significantly contributes to enhancing sports performance (Dobbs et al., 2019; Hao et al., 2019; Zhuying et al., 2020). The ability to sprint effectively often determines the outcome of competitions during critical moments (Liang et al., 2020). The warm-up phase is a critical component for optimizing the activation of explosive muscles. An effective warm-up not only increases body temperature and reduces the viscosity of muscle fibers but also enhances the range of motion of muscles and joints, thereby minimizing muscle damage. Additionally, it activates neural excitability, leading to improved athletic performance (Sener et al., 2021).

In the 1980s, Ramsey and Street (1941) proposed the phenomenon of Post-Activation Potentiation (PAP), which refers to the sustained increase in muscle contraction force following a period of intense voluntary contraction (Botelho and Cander, 1953). This is due to the strong neural impulses generated in the cerebral cortex by high-intensity voluntary contractions, leading to the phosphorylation of myosin regulatory light chains and increased calcium sensitivity of the actomyosin complex, thereby enhancing muscle strength (Vandervoort et al., 1983; Tillin and Bishop, 2009; Seitz and Haff, 2016). PAP has become a focal point in strength training. However, improvements in performance can only be observed when the PAP level reaches its peak value (approximately 150%) after the conditioning intervention. In fact, performance improvements may occur even in the absence of PAP (Blazevich and Babault, 2019). Recently, researchers have proposed the phenomenon of Post-Activation Performance Enhancement (PAPE), which may be related to residual effects of PAP following the conditioning intervention and other factors (Cuenca-Fernández et al., 2017; Boullosa et al., 2020). While the PAP enhancement effect has a relatively short duration (It is usually less than 3 min), the peak enhancement in muscle voluntary contraction force usually occurs between 6 and 10 min after the conditioning activity (Vandervoort et al., 1983; Cuenca-Fernández et al., 2017). Therefore, researchers believe that the increase in muscle strength beyond 4 min is attributed to PAPE (Vandervoort et al., 1983; Docherty and Hodgson, 2007; Cuenca-Fernández et al., 2017; Xie et al., 2022). This suggests that PAPE is more relevant than PAP in acute performance enhancement (Blazevich and Babault, 2019). Furthermore, the improvement in muscle strength depends on the relationship between enhancement and fatigue (Rassier and Macintosh, 2000). Numerous activation methods can generate significant neural impulses (e.g., electromyographic signals) and enhance performance in activities such as jumping, throwing, and sprinting (Seitz and Haff, 2016). Methods that induce PAPE include electrical stimulation, resistance training, accentuated eccentric loading, and sprint training (Seitz and Haff, 2016).

Some studies suggest that plyometric training, as opposed to traditional resistance training, may result in higher lower limb explosive strength following activation (Hughes et al., 2016; Bridgeman et al., 2017). Plyometric training utilizes the stretch-shortening cycle (SSC) principle to improve neuromuscular function and is commonly used to enhance lower limb strength (Hakkinen et al., 1985; Fatouros et al., 2000; Markovic et al., 2007), explosiveness, and other muscle functions (Brown et al., 1986; Matavulj et al., 2001; Salonikidis and Zafeiridis, 2008; Stanley, 2017). Experimental evidence indicates that plyometric training also promotes neuromuscular adaptations and increases muscle strength (Witzke and Snow, 2000; Kato et al., 2006). However, the effectiveness is influenced by factors such as individual training experience, recovery time, and the use of additional loads during the plyometric exercises. Higher training volume and intensity can lead to greater enhancement effects and fatigue levels (Tillin and Bishop, 2009), therefore, a balance between intensity-power complex and individual characteristics, such as training volume, intensity, and rest intervals, needs to be maintained between fatigue and enhancement (Seitz and Haff, 2016).

Previous research has indicated that plyometric training has a positive effect on lower limb strength (Markovic and Mikulic, 2010; Chen et al., 2023; Saez de Villareal et al., 2023).Higher training volume and intensity can result in greater enhancement effects and fatigue levels (Tillin and Bishop, 2009). However, there is no uniform conclusion on the effects of different forms of plyometric training on sprint ability and optimal interval time. Therefore, this study aims to investigate the impact of different combinations of plyometric training (single/complex forms, single set/multiple sets) on immediate vertical jump sprint performance. We categorized the rest intervals as short (0.3–4 min), moderate (5–7 min), and long (>8 min) (Seitz and Haff, 2016). This research may be helpful for jumping or short distance sprint athletes or people with sports experience to scientifically and accurately use plyometric training to improve sports performance and avoid unnecessary fatigue, especially for athletes to use different forms and different amounts of plyometric training in season or non-season, which has important practical significance.

## Methods

### Search strategy

**FIGURE 1:**
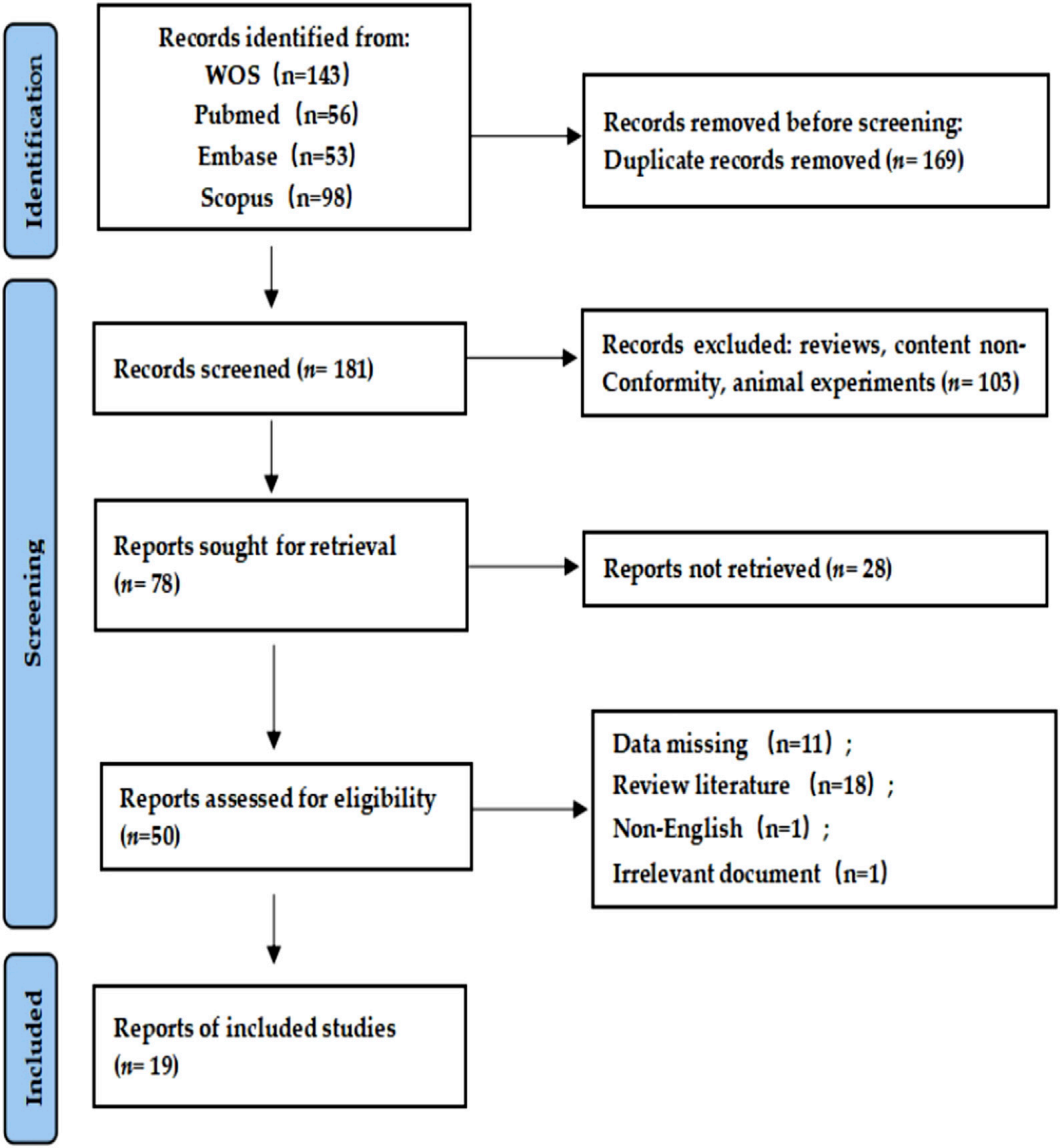
Literature screening flow chart.

The literature search was conducted independently by two individuals using a double-blind approach, following the PICOS principles. As shown in Figure 1, four databases, namely Web of Science, PubMed, Embase, and Scopus, were searched from their inception until 9 July 2023. A mixed combination search strategy was employed, using keywords such as “post-activation potentiation,” “plyometric,” “stretch shortening exercise,” “post-activation performance enhancement,” and “PAPE” for foreign language literature. Additionally, a secondary search of the references cited in the retrieved articles was performed to maximize the inclusion of relevant literature. A total of 350 articles were identified, with 143 from Web of Science, 56 from PubMed, 53 from Embase, and 98 from Scopus. We also used JASP 0.18.3 software to perform precision-effect test and precision-effect estimate with standard errors (PET-PEESE) to quantify reported publication bias and make appropriate corrections. If the PET effect-size estimate is significant, the PEESE model, specifying a weighted least square regression predicting the effect sizes with standard errors squared, is used for publication-bias adjustment because it provides a better effect-size approximation in the presence of an effect. If the PET effect-size estimate is not significant, the PET model and its effect-size estimate is usedAfter removing duplicate records using EndNote X9 (Bld 12062), 181 articles remained. Through the evaluation of titles and abstracts, irrelevant articles were excluded, leaving 78 articles for full-text review. After further assessment, 50 articles were deemed irrelevant, and a final set of 19 eligible articles were included for analysis.

### Inclusion and exclusion criteria

#### Inclusion criteria

Literature Type: The included literature consisted of randomized controlled trials (RCTs). Participants: Participants were not restricted by nationality but had a minimum of 1 year of sports experience. Intervention: The intervention measure applied was post-activation potentiation (PAP) training. Outcome Measures: The outcome measures assessed were countermovement jump (CMJ) performance and 20-meter sprint times.

#### Exclusion criteria

Excluded literature consisted of the following: duplicate articles, review articles, interventions other than post-activation potentiation (PAP) PAPE training, studies with missing data, participants with less than 1 year of sports experience, and non-English literature. Please refer to the Figure 1 for the specific process.

### Methodological quality evaluation

**FIGURE 2:**
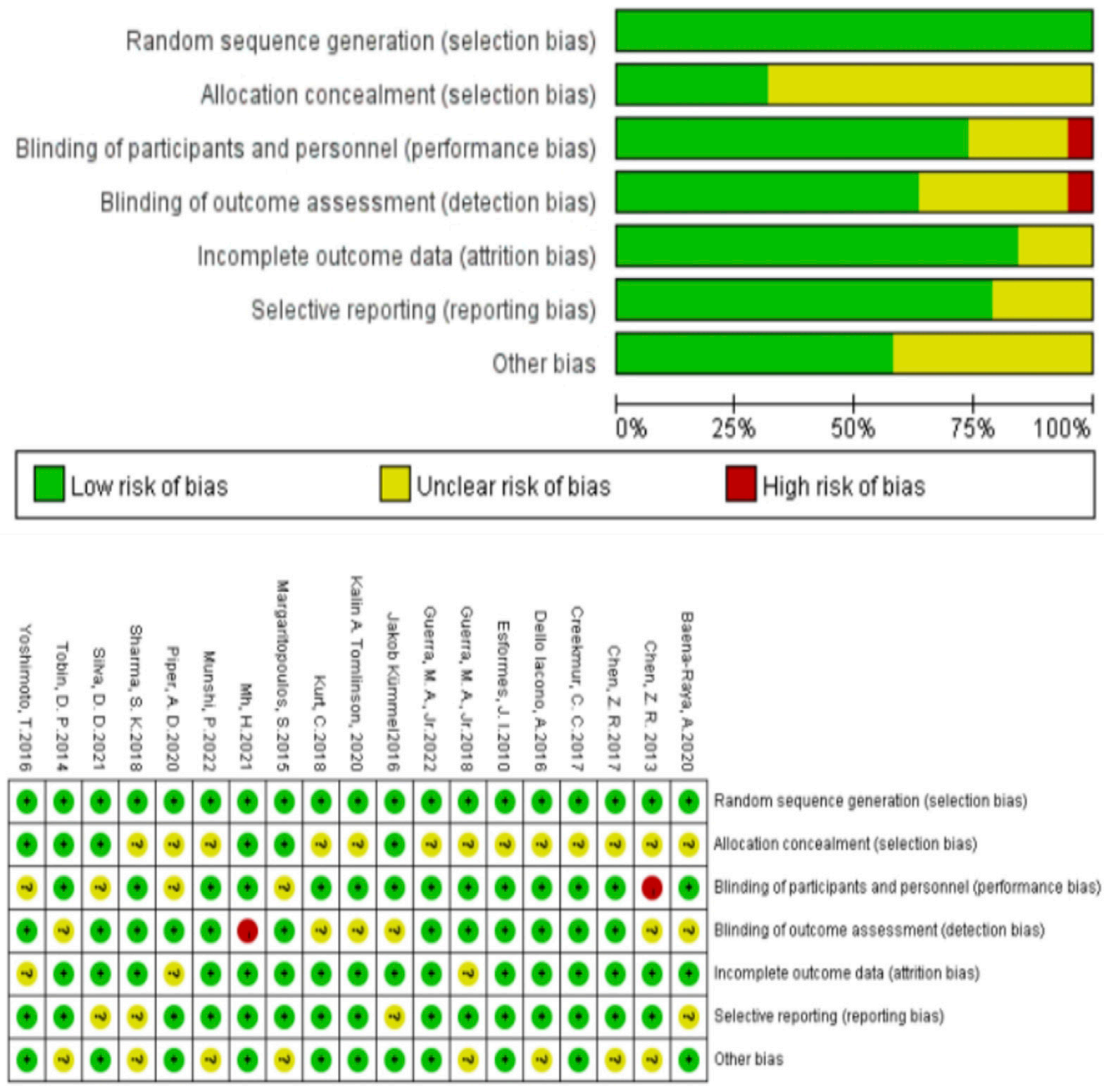
Risk bias assessment.

As shown in Figure 2 the quality of the included studies was assessed using the Cochrane Handbook for Systematic Reviews of Interventions version 5.1.0. The following criteria were evaluated: random sequence generation, allocation concealment, blinding of participants and personnel, blinding of outcome assessment, selective reporting, incomplete outcome data, and other sources of bias. The risk of bias for each included study was judged as high (C), unclear (B), or low (A). The methodological quality of the studies was rated as follows: C ≤ 1, 2 ≤ B ≤ 3, A ≥ 4. Five studies included participants with different levels of physical activity (n = 338). The methodological quality of the 19 included studies was assessed using the Cochrane risk of bias tool. As shown in Table 3.

### Data extraction

Two independent reviewers extracted data from the included studies using a standardized data extraction form. Data were extracted after full-text review. For data presented in graphs or figures that could not be directly extracted, the GetDate software was used. Extracted data included: first author, year of publication, sample size, participants’ sport, age, potentiation intervention, potentiation load, rest interval, countermovement jump (CMJ) height, and 20-m sprint time.

### Statistical processing

Data from the included studies were mostly extracted directly from the text. Review Manager 5.4 software was used to analyze the outcome measures. As the outcome measures were continuous variables, the standardized mean difference (SMD) was used as the effect size measure. The effect size was interpreted as follows: |SMD| < 0.5, small effect; 0.5 ≤ |SMD| < 0.8, moderate effect; |SMD| ≥ 0.8, large effect. Heterogeneity was assessed using the I2 statistic. I2 < 40% indicated low heterogeneity, 40% ≤ I2 ≤ 70% indicated moderate heterogeneity, and I2 > 70% indicated high heterogeneity. Fixed-effect models were used for studies with no heterogeneity or low heterogeneity, and random-effect models were used for studies with moderate or high heterogeneity. Subgroup analyses were conducted for studies with high heterogeneity.

### Publication bias analysis

**FIGURE 3:**
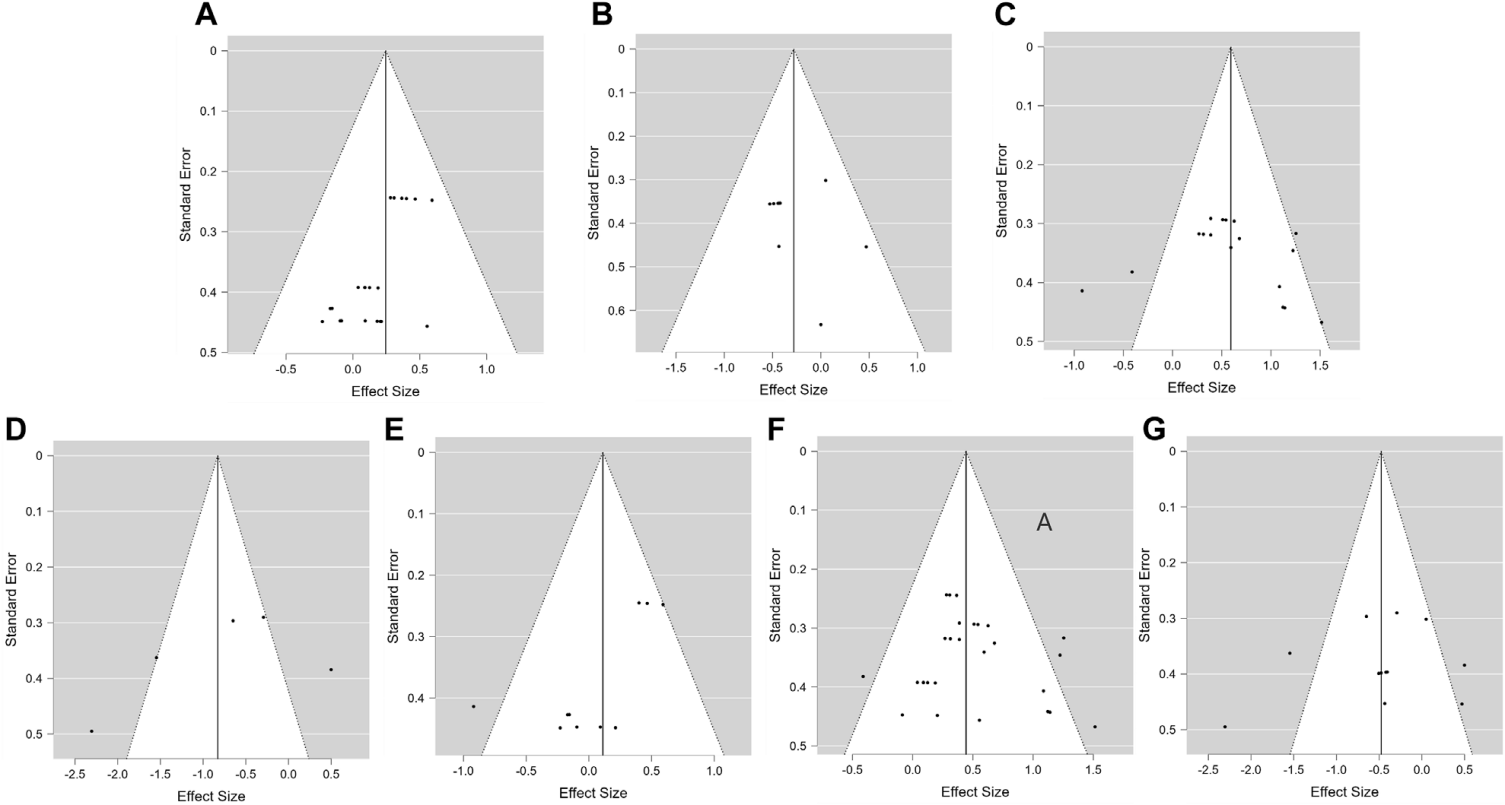
Funnel diagram of publication bias in the included literature.

The impact of PAP on jump performance was reported in 19 studies. Review Manager 5.4 was used to create a funnel plot for a qualitative analysis of publication bias in the included studies (Figure 3). The funnel plot shows that the literature points are evenly distributed on both sides of the centerline, indicating no significant publication bias.

Nineteen studies reported the effects of PAP on jump and sprint performance. Funnel plots were made using Review Manager 5.4 and Stata 15.0 to visually determine publication bias (Figure 3). The funnel plot showed that the reference points were evenly distributed on both sides of the center line, indicating no significant publication bias. To ensure scientific accuracy, we also used JASP 0.18.3 software to perform precision-effect test and precision-effect estimate with standard errors (PET-PEESE) to quantify reported publication bias and make appropriate corrections (Table 1). If the PET effect-size estimate is significant, the PEESE model, specifying a weighted least square regression predicting the effect sizes with standard errors squared, is used for publication-bias adjustment because it provides a better effect-size approximation in the presence of an effect. If the PET effect-size estimate is not significant, the PET model and its effectsize estimate is used (Stanley, 2017; Bartoš et al., 2022). As shown in Table 1, B (*P* = 0.564), C (P = 0.702), D (P = 0.394),F (0.755), G (P = 0.482) had no significant difference, indicating no publication bias. However, there was A significant difference between A (P = 0.001) and E (P = 0.008), indicating publication bias. PEESE corrected effect sizes were therefore used for the analysis (see Table 2).

**TABLE 1:** Test of publication bias.

Project	t	Df	p
A	−4.788	20	0.001
B	0.605	7	0.564
C	−0.390	15	0.702
D	−0.993	3	0.394
E	−3.497	8	0.008
F	0.316	27	0.755
G	−0.728	11	0.482

Note: A, Influence of single plyometrics training on vertical jumping ability; B, Influence of single plyometric training on sprint ability; C, Influence of complex plyometric training on vertical jumping ability; D, Influence of complex plyometric training on sprint ability; E, Influence of Single-session plyometric training on vertical jumping ability; F, Influence of multiple plyometrics training on vertical jumping ability; G, Influence of multiple plyometrics training on sprint ability.

**TABLE 2:** Estimates of PEESE.

	Estimate	Standard error	t	Df	95% confidence interval		
					p	Lower	Upper
A	0.457	0.035	8.127	20	0.001	0.370	0.527
E	0.475	0.191	3.952	8	0.06	0.381	1.132

## Results

### Study characteristics

Nineteen studies were included in the analysis following the PRISMA reporting guidelines, comprising a total of 19 randomized controlled trials. A total of 293 participants were included, with 163 males (55.6%), 21 females (7.2%), and 109 participants (37.2%) for whom gender was not reported. All participants had a training experience of more than 1 year and were part of the athletic population (Table 3).

**TABLE 3:** Included literature information table.

Name of document	Sample size	Means of intervention	Forms of intervention		Interval time (min)			Volume (set)		Index
			Single	Complex	0.3–4	5–7	≥8	1	≥2	
Esformes et al. (2010)	13	1*6 (speed bounds + right leg speed hops + left leg speed hops + vertical bounds)		✓		5		✓		CMJ
Sharma et al. (2018)	14	2*10 (ankle hops, three sets offive hurdle hops + five drop jumps from 50 cm height)		✓	1		10		✓	CMJ, 20M
Creekmur et al. (2017)	10	2 *8 plate jumps	✓			5			✓	40M
Munshi et al. (2022)	24	5*10 (legged vertical)+2*15 m (broad jumps)+1*30 m (legged bounding) +1*5 (depth jumps)		✓	4		12		✓	CMJ, 20M
Guerra et al. (2018)	24	2*15 (ankle hops)+ 3*5 (hurdle hops)+3*20 m (sprints with sled towing)		✓	1、3	5			✓	CMJ
Tomlinson et al. (2020)	22	2*8 (loaded squat jumps) 13%	✓			5			✓	20M
Piper et al. (2020)	13	Weighted Jump of max voluntary + 10% body weight	✓		4		8、12、16、20		✓	20M\CMJ
Chen et al. (2013)	10	3*5 (drop jump height)	✓			5		✓		CMJ
Guerra et al. (2018)	12	2 *15 (ankle hops)+ 3*5 (hurdle hops)+3*20 m (sprints with sled towing)		✓	1、3	5			✓	CMJ
Dello Iacono et al. (2016)	18	3*5 (vertical or horizontal-alternate one-leg drop jumps landing from a platform 25-cm		✓			8		✓	CMJ
Tobin and Delahunt (2014)	20	2*10 (ankle hops)+3*5 (hurdle hops)+1*5 (drop jumps from a height of 50 cm)		✓	1、3	5			✓	CMJ
Mh et al. (2021)	10	2*10 (ankle hops)+3*5 (hurdle hops)+1*5 (drop jumps)	✓				10		✓	CMJ
Hamsa et al., 2021	20	2*10 (ankle hops)+3*5 (hurdle hops)+1*5 (drop jumps from 50 cm)		✓	1	5			✓	CMJ, 20M
Silva et al. (2021)	11	4 continuous single-leg vertical jump for each leg	✓		1、3		8	✓		CMJ
Kurt et al. (2018)	13	4*5 (drop jump)or 2*10 (drop jump 30 cm)	✓		2				✓	CMJ
Baena-Raya et al. (2020)	34	3*5 (drop jump) or 1*5 (drop jump 50 cm)	✓		4		8、12	✓		CMJ
Kümmel et al. ()2016	5	10 reactive hops	✓				10	✓		20M
Yoshimoto et al. (2016)	10	3*10 hurdles (height 22 cm 、spaced 90 cm apart)	✓				10		✓	20M
Chen et al. (2013)	10	2*5 drop jump or drop jump	✓		2	6	12	✓		CMJ

### Influence of different forms of plyometric training on vertical jumping and sprinting ability

#### Influence of single plyometrics training on vertical jumping ability

**FIGURE 4:**
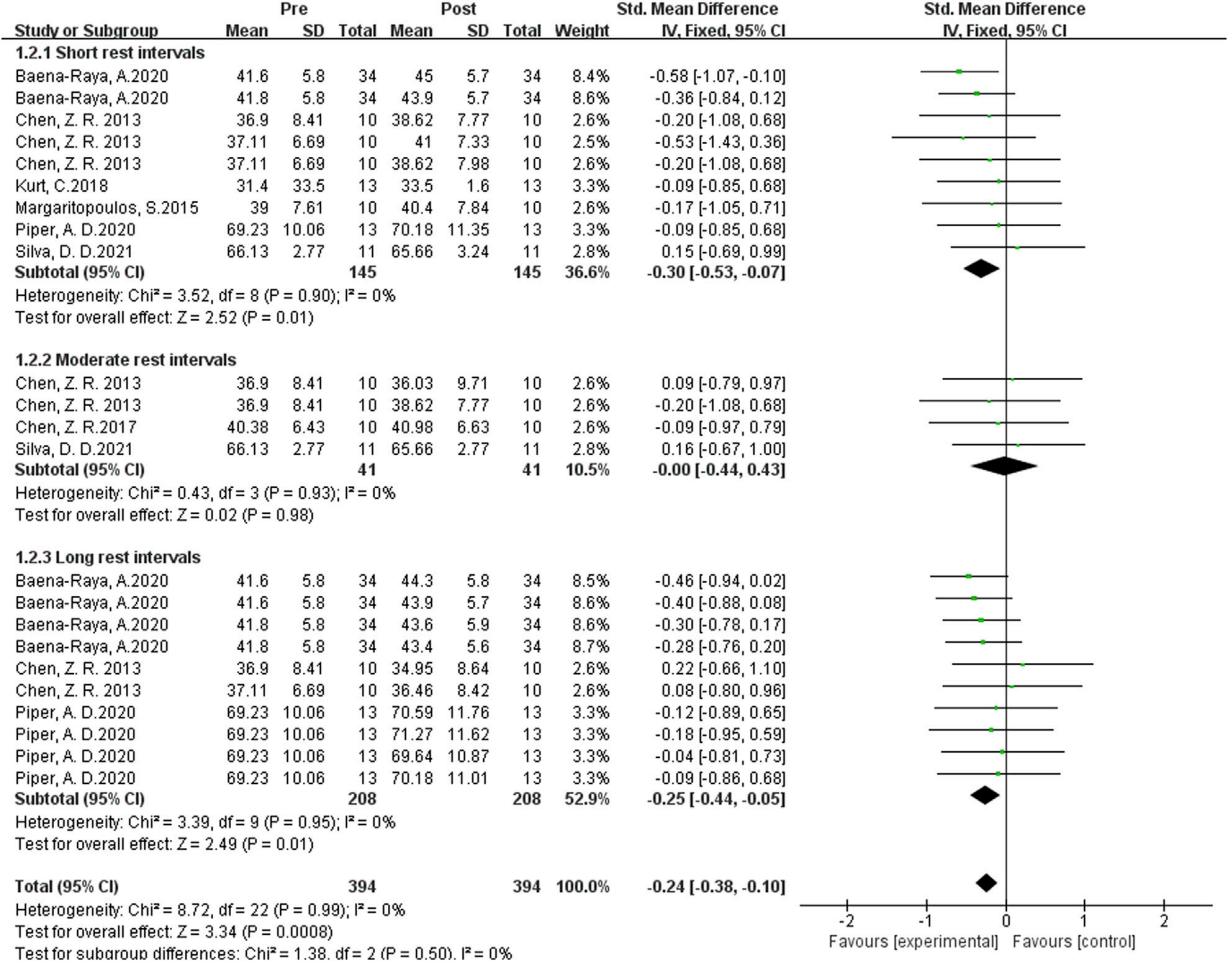
Subgroup analysis of the effects of single plyometric training on vertical jumping ability.

As shown in Figure 4 the data from 22 studies demonstrated the effects of single post-activation potentiation (PAP) training on vertical jump performance. Heterogeneity analysis revealed no statistical heterogeneity (I2 = 0 ≤ 40%, P = 0.0009), indicating the absence of significant heterogeneity among the studies. Therefore, a fixed-effects model was used for the meta-analysis of effect sizes, as shown in the figure. The combined effect size |SMD| = 0.24 > 0 indicates a small effect size, suggesting that single PAP training induces a slight improvement in vertical jump performance. [SMD = 0.045, 95% CI (−0.370, 0.527), P = 0.01].

Subgroup analysis results demonstrated that the largest effect size occurred with rest intervals of 0.3-4 min, with |SMD| = 0.30, P = 0.0008, indicating the most optimal enhancement effect. The effect size was slightly lower when the rest interval exceeded 8 min, with |SMD| = 0.25, indicating a mild activation effect. When the rest interval was between 5 and 7 min, the diamond symbol crossed the null line, suggesting no enhancement effect. In summary, when using single PAP training as the activation method, shorter rest intervals are more effective than longer rest intervals, while setrate rest intervals have little to no activation effect.

#### Influence of single plyometric training on sprint ability

**FIGURE 5:**
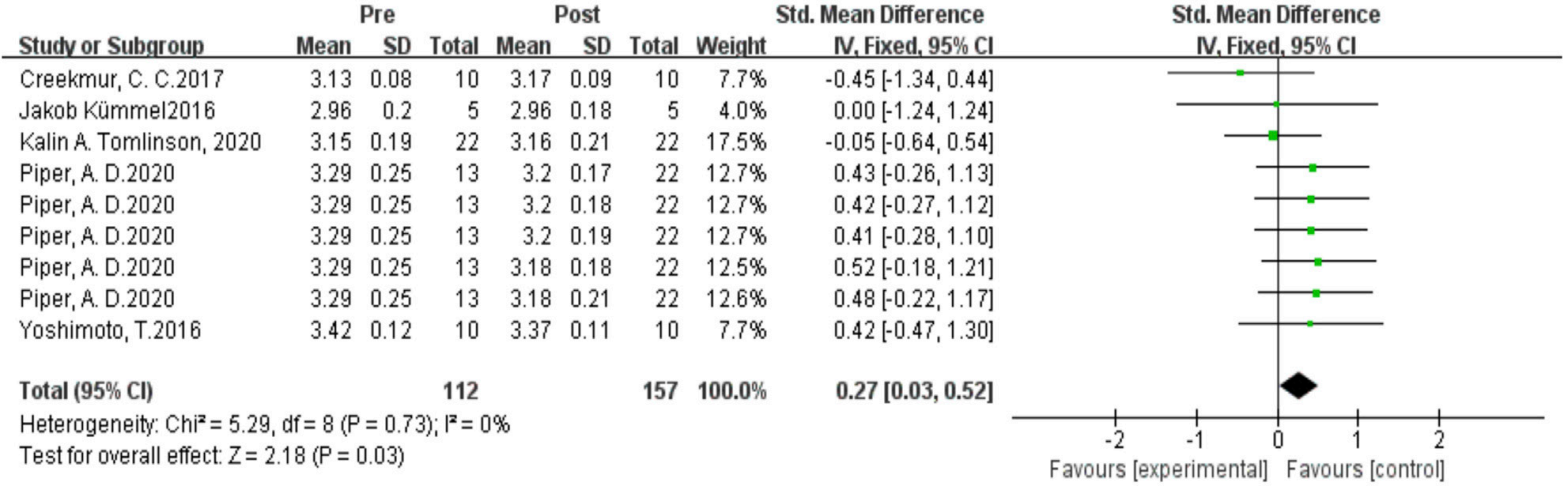
Effect of single plyometric training on sprint ability.

As shown in Figure 5 the data from 9 studies demonstrated the effects of single post-activation potentiation (PAP) training on sprint performance. Heterogeneity analysis revealed no statistical heterogeneity (I^2^ = 0 ≤ 40%, P = 0.03), indicating the absence of significant heterogeneity among the studies. Therefore, a fixed-effects setl was used for the meta-analysis of effect sizes, as shown in the figure. The combined effect size |SMD| = 0.27 > 0 indicates a small effect size, suggesting that single PAP training induces a slight improvement in sprint performance. [SMD = 0.27, 95% CI (0.03, 0.52), P = 0.03].

#### Influence of complex plyometric training on vertical jumping ability

**FIGURE 6:**
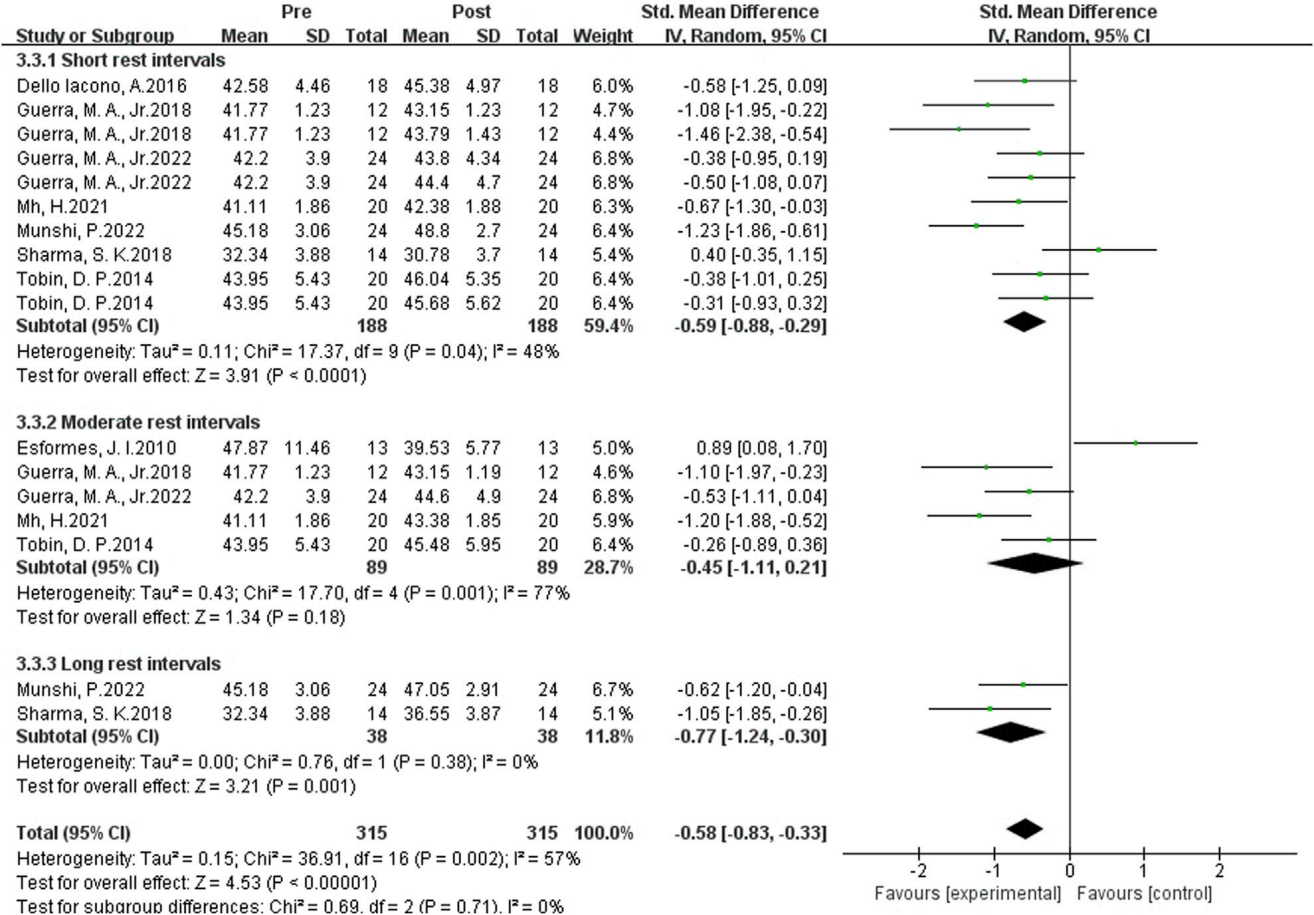
Subgroup analysis of the effects of complex plyometric training on vertical jumping ability.

As shown in Figure 6 the data from 17 studies demonstrated the effects of complex post-activation potentiation (PAP) training on vertical jump performance. Heterogeneity analysis revealed setrate statistical heterogeneity (I^2^ = 70% > 57%>40%, P = 0.002), indicating significant heterogeneity among the studies. Therefore, a random-effects setl was used for the meta-analysis of effect sizes, as shown in the figure. The combined effect size |SMD| = 0.58 > 0.5 indicates a setrate effect size, suggesting that complex PAP training induces a slight to setrate improvement in vertical jump performance. [SMD = 0.58, 95% CI (−0.86, −0.23), P = 0.002]. However, due to the high heterogeneity, further subgroup analysis is needed.

Subgroup analysis revealed substantial heterogeneity in the studies examining the effects of PAP on vertical jump performance. The results suggest that the source of heterogeneity may be the rest intervals. Studies with longer rest intervals showed the highest homogeneity (I^2^ = 0%), with the largest effect size (SMD = −0.77). Studies with setrate rest intervals (I^2^ = 77%) and short rest intervals (I^2^ = 48%) exhibited higher heterogeneity, with effect sizes of SMD -0.45 and −0.59, respectively. This suggests that the optimal rest interval for complex PAP training to induce the best enhancement effect on vertical jump performance is a longer interval, specifically greater than 8 min. When the rest interval exceeded 8 min, the effect size was the largest, with |SMD| = 0.77, indicating the best enhancement effect. When the rest interval was 0.3-4 min, the effect size was slightly lower, with |SMD| = 0.59, indicating a mild activation effect. When the rest interval was 5–7 min, the diamond symbol crossed the null line, suggesting no significant enhancement effect.

In summary, when using complex PAP training as the activation method, longer rest intervals are more effective than shorter rest intervals, while setrate rest intervals have little to no activation effect on vertical jump performance.

#### Influence of complex plyometric training on sprint ability

**FIGURE 7:**
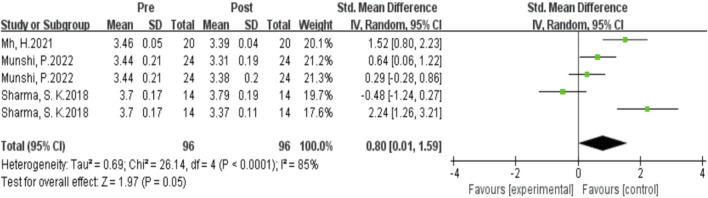
Subgroup analysis of the effect of single plyometrics training on longitudinal jumping ability

As shown in Figure 7 the data from 5 studies demonstrated the effects of complex post-activation potentiation (PAP) training on sprint performance. Heterogeneity analysis revealed high statistical heterogeneity (I^2^ = 85% > 70%, P = 0.05), indicating significant heterogeneity among the studies. Therefore, a fixed-effects setl was used for the meta-analysis of effect sizes, as shown in the figure. The combined effect size |SMD| = 0.8 ≥ 0.8 indicates a substantial effect size, suggesting that complex PAP training induces a significant improvement in sprint performance. [SMD = 0.8, 95% CI (0.01, 1.59), P = 0.05]. However, due to the high heterogeneity, further subgroup analysis is needed.

**FIGURE 8:**
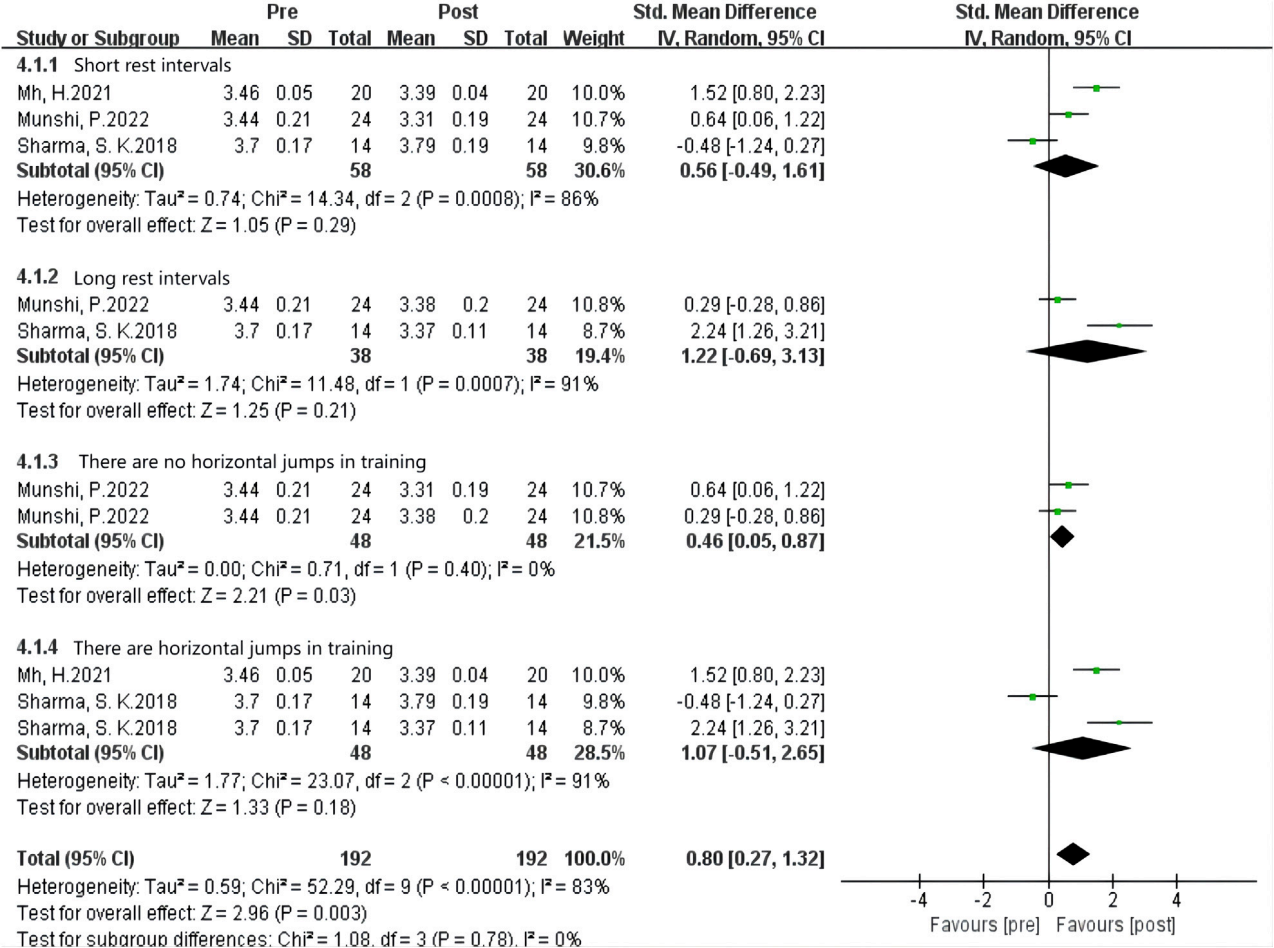
Subgroup analysis of the effects of complex plyometric training on sprint ability.

As shown in Figure 8 the subgroup analysis results indicate that the source of heterogeneity may be the intervention method. The studies involving horizontal jump exercises within the complex intervention training showed the highest homogeneity (I^2^ = 0%) and a setrate effect size (SMD = 0.46). On the other hand, the studies without horizontal jump exercises in the complex intervention training exhibited high homogeneity (I^2^ = 91%) and an effect size of SMD = 0.80. Regarding the rest intervals, whether short or long, there was high heterogeneity.

### Influence of different load capacities on vertical jumping and sprinting ability

#### Influence of single-session plyometric training on vertical jumping ability

**FIGURE 9:**
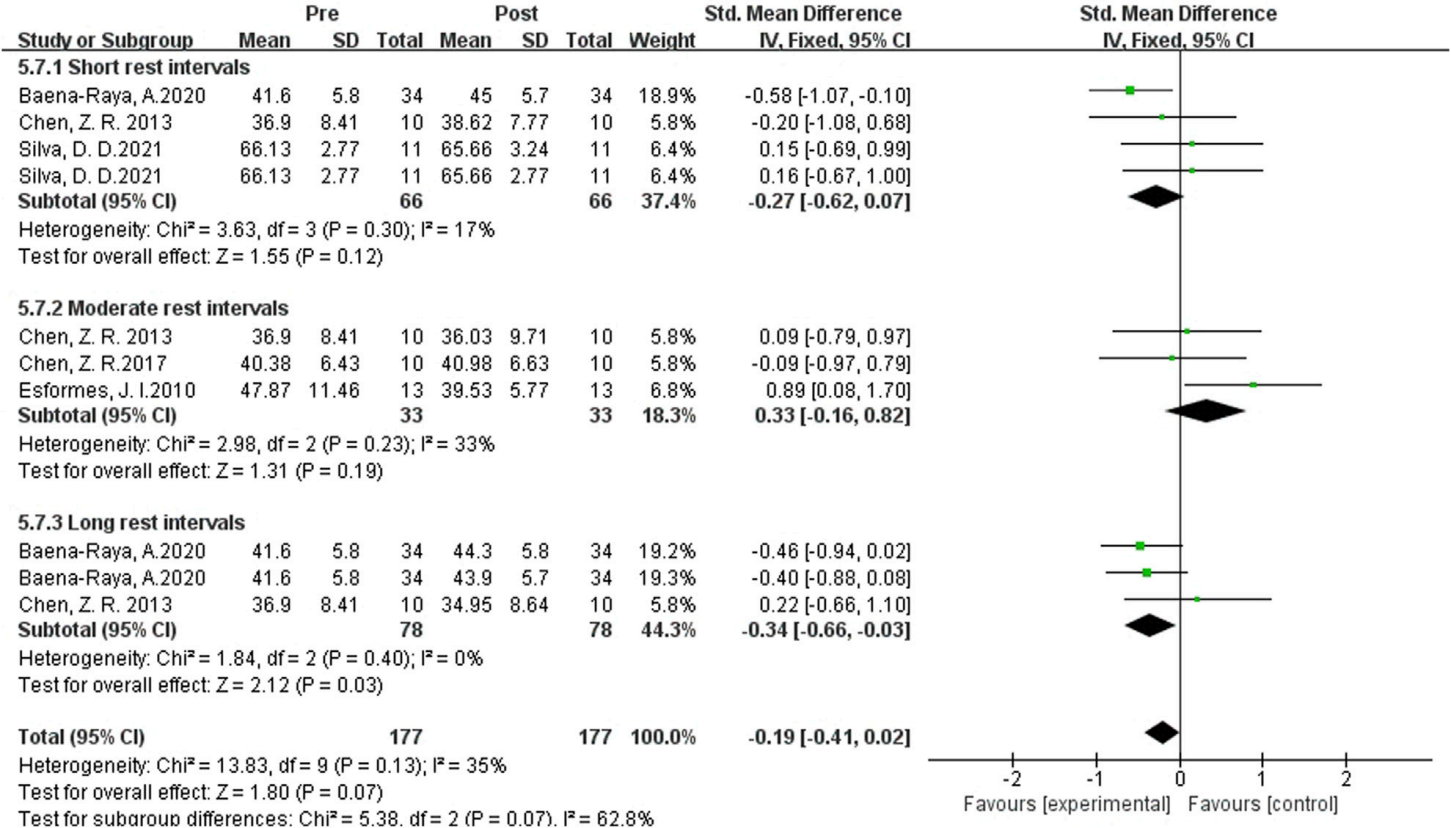
Subgroup analysis of the effects of a Single-session of plyometric training on vertical jumping ability.

As shown in Figure 9 the data from 10 studies examined the effects of single-set post-activation potentiation (PAP) training on vertical jump performance. It showed low statistical heterogeneity (I^2^ = 35% < 40%, P = 0.07), indicating a relatively low level of heterogeneity among the studies. Therefore, a fixed-effects setl was used for the meta-analysis of effect sizes. The diamond symbol crossed the null line, and the p-value was greater than 0.05, suggesting that single-set PAP training has no significant improvement on vertical jump performance. [SMD = −0.475, 95% CI (0.381, 1.132), P = 0.06].

Subgroup analysis examined the effects of complex PAP training on vertical jump performance. When the rest interval exceeded 8 min, the effect size was the largest with |SMD| = 0.77, indicating the best enhancement effect. When the rest interval was 0.3-4 min and 5–7 min, the diamond symbol crossed the null line, suggesting no significant enhancement effect. When the rest interval exceeded 8 min, the effect size was |SMD| = 0.34, indicating a slight improvement in vertical jump performance.

In summary, when using single-set PAP training as the activation method, only long rest intervals have an enhancement effect, while short and setrate rest intervals do not have a significant enhancement effect on vertical jump performance.

#### Influence of multiple plyometrics training on vertical jumping ability

**FIGURE 10:**
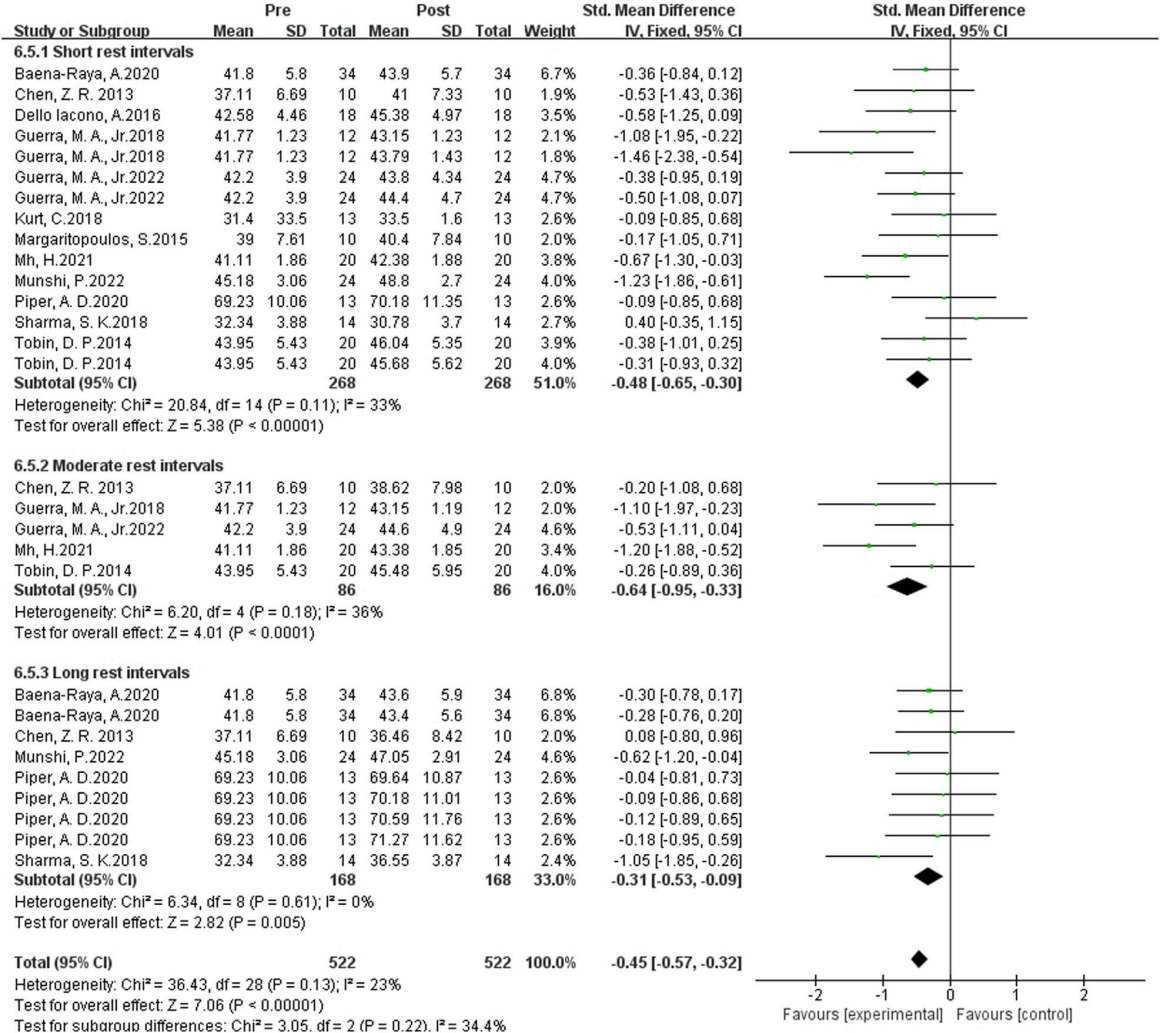
Subgroup analysis of the effects of multiple groups of plyometric training on vertical jumping ability.

As shown in Figure 10 the data from 29 studies examined the effects of complex post-activation potentiation (PAP) training on sprint performance. Heterogeneity analysis showed a high level of statistical heterogeneity (I^2^ = 23% ≤ 40%, P = 0.00001 < 0.05), indicating significant heterogeneity among the studies. Therefore, a fixed-effects setl was used for the meta-analysis of effect sizes, as shown in the figure. The combined effect size |SMD| = 0.43 > 0, indicating a significant effect size, suggesting that complex PAP training induces an improvement in sprint performance. [SMD = 0.43, 95% CI (0.01, 1.59), P = 0.00001 < 0.05].

Subgroup analysis examined the effects of Multiple-session PAP training on sprint performance. When the rest interval was 5–7 min, the effect size was the largest with |SMD| = 0.64, indicating the best enhancement effect. When the rest interval was 0.3-4 min and greater than 8 min, the effect sizes were |SMD| = 0.48 and 0.31, respectively, indicating a slight improvement in sprint performance during these two time intervals.

In summary, when using Multiple-session PAP training as the activation method, setrate rest intervals are more effective than short rest intervals, while long rest intervals have the least activation effect on sprint performance.

#### Influence of multiple plyometrics training on sprint ability

**FIGURE 11:**
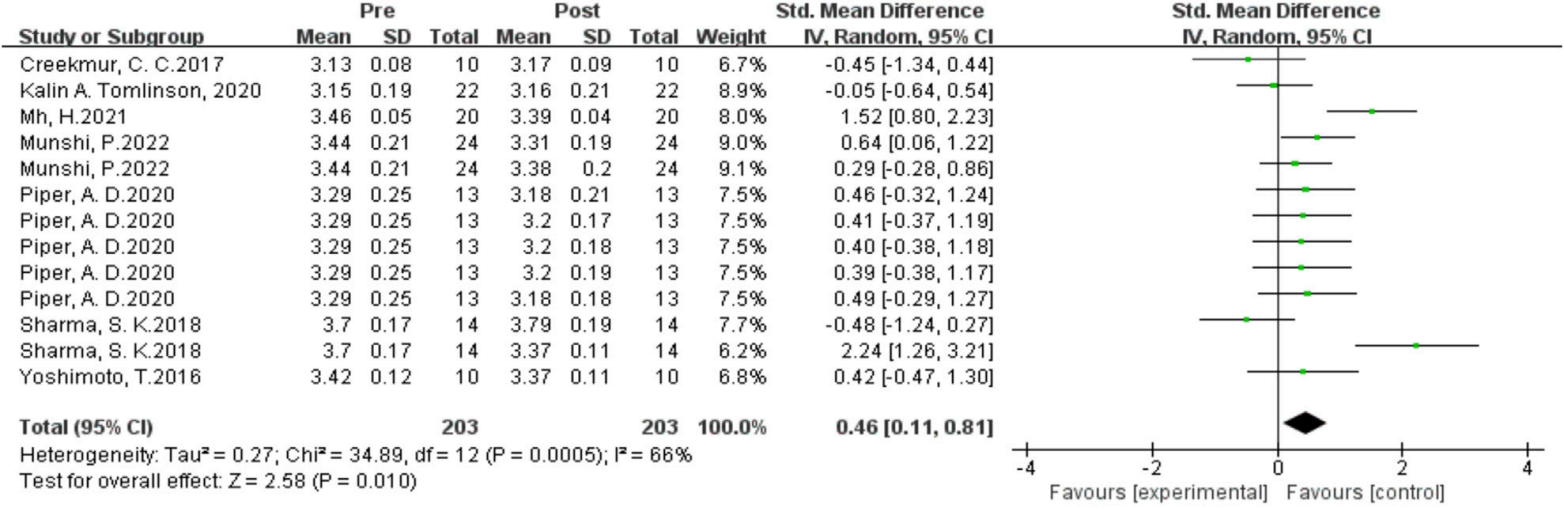
Effects of multiple plyometrics training on sprint ability.

As shown in Figure 11 the data from 13 studies examined the effects of Multiple-session post-activation potentiation (PAP) training on sprint performance. Heterogeneity analysis showed a high level of statistical heterogeneity (I^2^ = 66%, P = 0.01 < 0.05), indicating significant heterogeneity among the studies. Therefore, a random-effects setl was used for the meta-analysis of effect sizes, as shown in the figure. The combined effect size |SMD| = 0.46 > 0, indicating a significant effect size, suggesting that Multiple-session PAP training induces an improvement in sprint performance. [SMD = 0.46, 95% CI (0.01, 0.81), P = 0.01 < 0.05].

**FIGURE 12:**
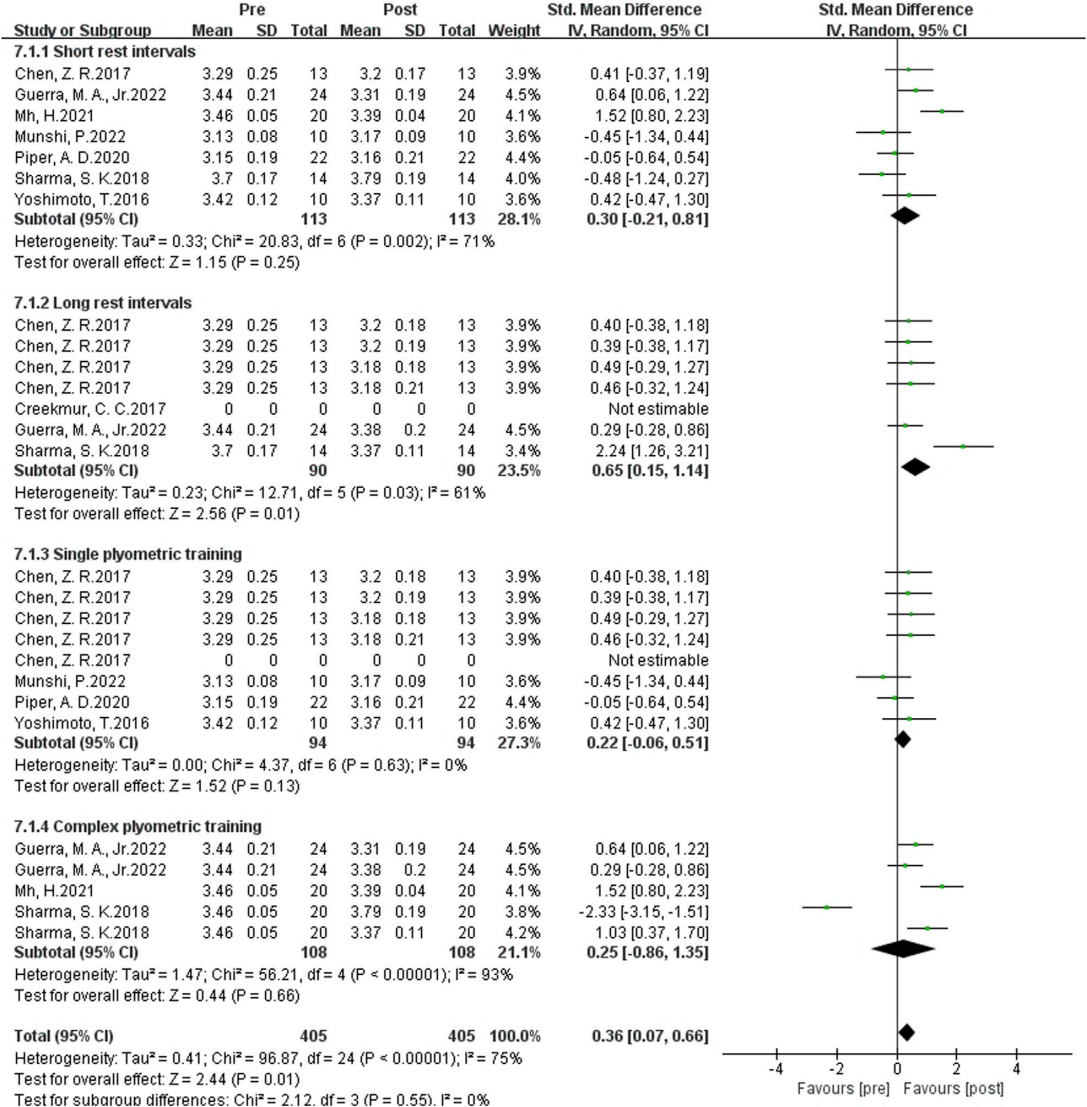
Subgroup analysis of the effects of plyometric training on sprint ability.

As shown in Figure 12 Subgroup analysis results showed that the rest interval had significant heterogeneity, and the source of heterogeneity may be the different types of enhancement interventions. Single-set training had the highest homogeneity (I^2^ = 0%) and the largest effect size (SMD = 0.22). Studies on multiple plyometrics training showed higher heterogeneity (I^2^ = 93%) with an effect size of SMD = 0.25, indicating that multiple plyometrics training has the best enhancement effect on sprint performance with longer rest intervals, specifically greater than 8 min.

## Discussion

### Influence of different forms of plyometric training on vertical jumping and sprinting ability

#### Influence of single plyometrics training on vertical jump and sprint ability

Meta-analysis results indicate that Single-session plyometric training induces a modest enhancement in vertical jump performance in athletic populations. This is consistent with the findings of (Gil et al., 2019; Gil et al., 2020). Optimal jump performance is observed with short rest intervals, followed by long intervals, while moderate intervals yield minimal improvements. The enhancement effect is influenced by two critical “windows of opportunity” (Meifu and Guo, 2019).

Post-intervention, muscle fatigue and potentiation mechanisms coexist, with their balance affecting power output and overall performance. Single-set interventions elicit a limited potentiation effect due to their singular nature. In the short term, potentiation dominates, with fatigue levels below activation levels, resulting in elevated power output during the “first window of opportunity.” As rest intervals increase, the potentiation effect diminishes, fatigue becomes more pronounced, and vertical jump performance suffers. With further of rest intervals, fatigue dissipates, and the potentiation effect once again becomes dominant, leading to improved power output during the “second window of opportunity.” Plyometric exercises generate elevated muscle temperatures, enhancing muscle activation and improving athletic performance, tendon tissue storage, and recoil capacity (Castro-Garrido et al., 2020; Mh et al., 2021).

Single-session plyometric training has been shown to induce modest improvements in sprint performance in athletic populations. Due to the relatively low stimulus of Single-session plyometric training, Creekmur et al. (2017) utilized weighted ankle jumps as a potentiating exercise. Repetitive weighted potentiated jumps elicit sufficient central nervous system stimulation to enhance sprinting ability. Research by Piper et al. (2020) has demonstrated improved sprint speeds following potentiating exercises, with 8-min and weighted potentiated exercises resulting in faster sprint times compared to 4-min protocols.

The potentiation effect elicited by longer rest intervals is greater than that of shorter intervals, as fatigue can interfere with potentiation if the PAP stimulus occurs too close to the subsequent exercise. Yoshimoto et al. (2016) study showed that high-frequency small-hurdle running improved sprint performance, possibly due to increased stride frequency, which significantly enhances velocity during the acceleration phase of sprinting. Plyometric exercises such as hurdle hopping involve horizontal potentiating jumps, with movement patterns similar to those in sprinting, thus facilitating greater positive transfer and improving sprint performance (Kümmel et al., 2016). Performing multiple repetitions of single-set potentiating jumps is an effective method to harness the PAP phenomenon, providing sufficient potentiation without inducing excessive fatigue, which can minimize unnecessary interference from complex dynamic training protocols.

#### Influence of complex plyometric training on vertical jumping and sprinting ability

Meta-analysis results indicate that complex plyometric training induces a significant enhancement in vertical jump performance in athletic populations. Optimal jump performance is observed with long rest intervals, followed by short intervals, while moderate intervals yield minimal improvements, which is in contrast to the effects of Single-session plyometric training on vertical jump ability. Complex plyometric training interventions are more complex and involve greater intensity, thus eliciting higher levels of fatigue.

During short rest intervals, the potentiation effect is marginally stronger than the degree of fatigue, resulting in a modest improvement in performance. As the rest interval increases, fatigue gradually diminishes, but so does the potentiation effect, with fatigue becoming more dominant and vertical jump performance suffering. With further prolongation of the interval, fatigue is nearly eliminated, while the potentiation effect persists, leading to enhanced vertical jump performance. Plyometric exercises with slightly higher intensities are more effective in improving vertical jump ability (Esformes et al., 2010; Mh et al., 2021). Complex plyometric training interventions, which include exercises such as alternate-leg hopping and single-leg hopping, further increase the intensity.

Therefore, potentiating exercises such as single-leg or alternate-leg hopping and depth jumps are more effective in enhancing vertical jump performance, and complex plyometric training protocols are superior to Single-session plyometric training, offering a more suitable option for athletes requiring reduced training volume.

Complex plyometric training induces a significant enhancement in sprint performance in athletic populations, with a larger effect size compared to Single-session plyometric training. This is likely due to the higher intensity of complex training, which further increases neural excitation and potentiation. Sharma et al. (2018) study demonstrated that 20-meter sprint times were reduced 10 min after complex potentiating interventions compared to 1 min, indicating improved sprinting performance. The study also found that blood lactate levels were higher at 1 min post-intervention than at 10 min, with a corresponding decrease in 20-meter performance; however, as blood lactate decreased (at 10 min), 20-meter sprint times improved significantly. This suggests that immediately following the intervention, lactate accumulation in the blood leads to fatigue that outweighs the potentiation effect, resulting in decreased sprint performance; as the recovery period increases, lactate is broken down, fatigue is reduced, and the potentiation effect becomes dominant, leading to enhanced sprinting performance. Sharma et al.’s study showed an immediate 2.4% increase in sprint time after potentiation training, followed by an 8.9% decrease after a 10-min recovery, which is consistent with the findings of Sharma, S. K. and Mh et al. (2021). This may be related to increased phosphorylation of myosin regulatory light chains, enhanced recruitment of high-threshold motor units, and altered pennation angles (Meifu and Guo, 2019).

Subgroup analysis revealed that potentiation training interventions involving horizontal jumps resulted in superior sprint performance compared to those without horizontal jumps. This is likely because horizontal potentiating jumps generate a horizontal force vector, while vertical potentiating jumps produce minimal horizontal force, thus making the former more effective for sprint performance. From a mechanical perspective, the vertical and horizontal components of ground reaction force and the corresponding impulses are primary determinants of athletic performance. Studies have shown that horizontal jumps are more effective for increasing acceleration over short distances (less than 10 m), while vertical potentiation training is better suited for improving vertical jump performance (Tobin and Delahunt, 2014; Dello Iacono et al., 2016; Abade et al., 2017; Silva et al., 2021). The mechanism of potentiation should mimic the intended athletic performance as closely as possible to maximally stimulate the same neural pathways (Tobin and Delahunt, 2014).

### Influence of different load capacities on vertical jumping and sprinting ability

#### Influence of single-session plyometric training on vertical jumping ability

Single-session plyometric training induces no significant enhancement in vertical jump performance in athletic populations. Subgroup analysis revealed no improvement in vertical jump performance with short and moderate rest intervals, but a slight improvement was observed with long rest intervals (George et al., 2019).

During short and moderate rest intervals, the potentiation effect elicited by Single-session plyometric training is minimal, insufficient to recruit high-threshold fast-twitch muscle fibers and enhance postsynaptic potentials, while simultaneously inducing fatigue, leading to decreased vertical jump performance (Guerra et al., 2018; Sharma et al., 2018; Baena-Raya et al., 2020). As the rest interval increases, fatigue dissipates at a faster rate than the potentiation effect, resulting in improved vertical jump performance after long rest intervals. Single-session plyometric training, with its low training volume, does not induce PAP but rather PAPE, resulting in no improvement in vertical jump performance with short rest intervals and a slight improvement with long rest intervals. Performance enhancements (PAPE) can also occur in the absence of PAP, with peak potentiation effects occurring after longer rest intervals (Zimmermann et al., 2020). Esformes et al. (2010); Chen et al. (2013) study suggests that single-set potentiation training may not have elicited sufficiently high muscle fiber recruitment to enhance postsynaptic potentials, thereby generating an adequate potentiation effect. Vandervoort et al. (1983) study demonstrated that fatigue from muscle contractions exceeding 10 s can partially inhibit potentiation. The duration of potentiation training in Esformes, J. I.’s study was 70 s, which may have induced high levels of metabolic fatigue, interfering with the potentiation response and resulting in fatigue outweighing potentiation, thus limiting the improvement in vertical jump performance. Weighted potentiation exercises can rapidly achieve high levels of threshold motor unit activation with minimal fatigue, similar to the potentiation effect observed after high-intensity exercise, as the added weight increases the training intensity. Increasing training intensity is necessary when employing single-set potentiation training with reduced training volume (Mh et al., 2021).

#### Influence of multi-group plyometric training on vertical jumping and sprinting ability

Meta-analysis results indicate that Multiple-session plyometric training significantly enhances sprint performance in athletic populations. Furthermore, the potentiation effect is most pronounced with moderate rest intervals, followed by short intervals, and least with long intervals. Previous research has shown that fatigue dissipates faster than potentiation, and athletes with higher athletic ability exhibit greater CMJ performance, possibly due to their capacity to resist fatigue or recover from it more quickly (Guerra et al., 2022). Chen et al. (2013) suggested that Multiple-session plyometric training with fewer than 3 sets can effectively induce a potentiation effect while minimizing fatigue, thereby improving vertical jump performance. Margaritopoulos et al. (2015), in his study, demonstrated that a 5-min rest interval was sufficient to induce a potentiation effect and enhance vertical jump performance in highly trained elite karate athletes. Mh, H. also reported that CMJ height was improved to a greater extent 5 min after potentiation exercises compared to 1 min.

Therefore, we speculate that the reason for these findings is that Multiple-session interventions elicit a greater potentiation effect due to the higher training load, but they also induce higher levels of fatigue. Consequently, with short rest intervals, insufficient recovery from fatigue limits the enhancement of vertical jump performance compared to moderate rest intervals (Kurt et al., 2018). With long rest intervals, although fatigue dissipates rapidly, the potentiation effect also diminishes, resulting in the least improvement. This suggests that when implementing Multiple-session plyometric training, it is crucial to avoid excessive sets and to select rest intervals that are neither too long nor too short, optimizing the timing of the potentiation effect and fatigue recovery.

Multiple-session plyometric training also significantly enhances sprint performance in athletic populations. Subgroup analysis revealed that Multiple-session plyometric training has no effect on sprint performance with short rest intervals, but it does improve sprint performance with long rest intervals. Mh, H. reported that Multiple-session weighted plyometric training can improve sprint performance; however, due to the high training load and the addition of external weight, fatigue outweighs the potentiation effect in the short term (less than 4 min) following the intervention, resulting in no improvement in sprint performance (Piper et al., 2020; Munshi et al., 2022). After a period of recovery, fatigue diminishes, and the potentiation effect becomes dominant, leading to enhanced sprint performance. Some studies have suggested that sprint speed does not improve until 3 min after Multiple-session plyometric training, gradually increases after 5 min, and can persist for up to 12 min, while isometric strength gains may persist for up to 20 min. Creekmur et al. (2017) proposed that since ankle hopping is a relatively weak stimulus, they employed repeated weighted ankle jumps (2 sets of 8 repetitions) and hypothesized that this would be sufficient to induce a potentiation effect. Their study results demonstrated that Multiple-session potentiating jumps elicit sufficient central nervous system stimulation to enhance sprint performance with longer rest intervals. Our analysis suggests that the effectiveness of Multiple-session plyometric training for improving sprint performance depends on the rest interval and training load, and it is important to optimize the timing of the potentiation effect and fatigue recovery.

## Conclusion

The enhancing effects of Single plyometric training on vertical jump performance are maximized after short rest intervals, followed by long rest intervals, while there is almost no improvement after moderate rest intervals. Complex plyometric training demonstrates its greatest enhancement effect on vertical jump performance after long rest intervals, followed by short rest intervals, with no improvement observed after moderate rest intervals. Single-session plyometric training has a slight positive effect on sprinting ability, whereas the enhanced effects induced by complex plyometric training significantly improve sprinting ability.

Single-session plyometric training does not have a significant impact on vertical jump performance after short and moderate rest intervals, but it shows a slight improvement after long rest intervals. Multiple-session plyometric training has a significant enhancing effect on vertical jump performance, with the most notable enhancement observed after moderate rest intervals, followed by short rest intervals, while the effect is weakest after long rest intervals. Multiple-session plyometric training has a significant impact on sprinting ability, but there is no improvement observed after short rest intervals, while there is an improvement after long rest intervals.

### Practical application

Adding plyometrics to your routine or warm-up before a race to activate your body’s potential can improve performance later in the day. Plyometrics do not require complex or heavy exercise equipment, only need to overcome the body weight with your bare hands to complete. Based on the conclusions of this study, for people with training experience, we put forward the following suggestions: For the events with vertical jumping, if the training state is not good, we suggest to do 1 set of complex plyometric training and rest for 0.3–4 min before training or competition; If you feel good, do 2-4 sets of complex plyometrics and rest for 8–16 min before training or competing. For events with short sprints, we recommend 2-4 sets of complex plyometric training and 8–16 min rest if the training condition is good; If the training condition is not good, it is recommended to do 2-4 sets of single plyometric training and rest for 4–8 min before training or competition.

### Research deficiencies and prospects

The subjects of this study were individuals with a certain level of sports background, and thus the conclusions may not be applicable to individuals with no history of physical activity. The literature included in this study does not differentiate between post-activation potentiation (PAP) and post-activation performance enhancement (PAPE), which can lead to confusion when discussing the mechanisms of plyometric training on vertical jump and sprinting ability.

In future investigations of post-activation potentiation (PAP), it is advisable to utilize electromyographic systems to objectively assess its true effects. Currently, there is limited evidence regarding the effectiveness of post-activation potentiation (PAP) strategies in implementing plyometric training programs for high-level athletes. From a biomechanical perspective, the vertical and horizontal components of ground reaction forces (GRF) and their impulses are critical determinants of athletic performance. Future research should delve deeper into these aspects to design plyometric training programs in a more scientifically accurate manner.
